# Composite poplars: a novel tool for ectomycorrhizal research

**DOI:** 10.1007/s00299-017-2212-2

**Published:** 2017-10-23

**Authors:** Dimitri Neb, Arpita Das, Annette Hintelmann, Uwe Nehls

**Affiliations:** Faculty 2, Biology/Chemistry, Botany, University of Bremen, Leobener Str. 2, 28359 Bremen, Germany

**Keywords:** Agrobacterium, Plant transformation, Composite poplars, Ectomycorrhiza, Fluorescent proteins

## Abstract

**Key Message:**

**Composite poplars were used for ectomycorrhiza formation. Structurally normal mycorrhizas of transgenic roots revealed better fungal sugar support. Targeting fluorescent proteins to peroxisomes allowed easy in planta visualization of successful transformation**.

**Abstract:**

A bottle neck in ectomycorrhizal research is the time demand for generation of transgenic plants. An alternative strategy for such root-centered research might be the formation of the so-called composite plants, where transgenic roots are formed by non-transgenic shoots. We have developed an *Agrobacterium rhizogenes-*mediated root transformation protocol using axenic *Populus tremula* × *tremuloides* and *P. tremula* × *alba* cuttings. When comparing four different bacterial strains, *A. rhizogenes* K599 turned out to be the most suitable for poplar transformation. Transgenic roots revealed only minor hairy root phenotype when plants were grown on agar plates with synthetic growth medium in the absence of a sugar source. When using different ectomycorrhizal fungi, formation of ectomycorrhizas by transgenic roots of composite poplars was not affected and mycorrhizas were anatomically indistinguishable from mycorrhizas of non-transgenic roots. Elevated trehalose content and marker gene expression, however, pointed towards somewhat better fungal carbon nutrition in ectomycorrhizas of transgenic compared to non-transgenic roots. Cell wall autofluorescence of poplar fine roots is an issue that can limit the use of fluorescent proteins as visual markers for in planta analysis, especially after ectomycorrhiza formation. By targeting marker proteins to peroxisomes, sensitive fluorescence detection, easily distinguishable from cell wall autofluorescence, was obtained for both poplar fine roots and ectomycorrhizas.

## Introduction

Due to a frequently nutrient limited environment combined with extended seasonality, boreal and temperate forests dominate large parts of the northern hemisphere. To guarantee optimal function of these ecosystems, the mutualistic interaction of tree roots with ectomycorrhizal (ECM) fungi is essential (Smith and Read 2008). In this type of symbiosis, the fungal partner provides soil-based water and mineral nutrients, while the plant serves the basic carbohydrates from photosynthesis.

Due to its rapid growth and easily handling properties under laboratory and green house conditions, together with the establishment of molecular tools (Holsters et al. 1978) and *Agrobacterium tumefaciens*-based transformation protocols (e.g., Nowak et al. 2004; Ohmiya et al. 2003; Park et al. 2009; Zhang et al. 2014), poplar became a favored model for ectomycorrhizal research. With respect to ectomycorrhizal plant colonization, a (limited) number of laboratory- and field-based (e.g., Stefani et al. 2009; Danielsen et al. 2013) studies revealed no general impact of transgenic properties on symbiosis. However, due to the time request for generation and propagation of transgenic plants, which usually lasts for more than a year (Alpizar et al. 2006; Hampp et al. 1996), generation of transgenic poplars is still a bottleneck, limiting ECM research.

With the establishment of the so-called composite plants, where transgenic roots emerge from non-transgenic shoots after *Agrobacterium rhizogenes* infection, Hansen et al. (1989) established an alternative strategy for plant transformation. Generation of transgenic roots is much faster (a few weeks) than the conventional transformation (several month) for many plants (Veena and Taylor 2007). However, transgenic root induction by *A. rhizogenes* also has some drawbacks. Most obviously, generation of transgenic roots by non-transgenic shoots limits this approach mainly to root-centered questions, even if some examples of above-ground gene silencing in composite plants by dsRNA expression are found in the literature (Veena and Taylor 2007). A restriction of transgenic properties to roots has, however, also advantages. Interference with gene function in other plant organs is omitted in most cases.

A second difference to the conventional *A. tumefaciens*-based transgenic plants is that roots of composite plants frequently reveal a so-called hairy root phenotype, which is characterized by the extensive development of root hairs, and a reduced gravity response (Veena and Taylor 2007). Responsible for hairy root characteristics is the requirement of non-disarmed bacterial strains for root induction. These strains still contain specific Ri plasmids that harbor distinct T-DNA regions, which are transmitted together with a second T-DNA of interest (located on a shuttle vector) into plant cells (An 1985; Collier et al. 2005; Mankin et al. 2007). These Ri-plasmid located T-DNAs contain genes that affect either plant auxin response or lead to increased auxin biosynthesis (Cardarelli et al. 1987). Thereby they cause the induction of shoot-derived transgenic roots but also the so-called hairy root phenotype.

Despite these limitations, numerous successful applications of composite plants both in applied (Srivastava and Srivastava 2007; Banerjee et al. 2017) and basic research (see below) are found in the literature. *A. rhizogenes*-based approaches were used for gene expression analysis at cell type resolution (e.g., Ron et al. 2014) or investigations of mechanisms of gene regulation (e.g., Kim et al. 2011). Composite plants were used in particular to investigate root development (e.g., Kereszt et al. 2007), especially in response to biotic interaction with nematodes (e.g., Wubben et al. 2009), bacteria (e.g., Hirsch et al. 2009), or fungi (e.g., Mrosk et al. 2009).

To elucidate the potential of composite plants in ectomycorrhizal research and to speed up molecular and functional research of this type of symbiosis, we have developed an *A. rhizogenes*-based protocol for a fast generation of composite poplars and investigated the ability of transgenic roots to form functional ectomycorrhizas. *A. rhizogenes* strains that are commonly used for plant transformation differ in their Ri-plasmid and can be grouped by the type of opines (agropine type, mannopine type, cucumopine type, and mikimopine type) produced by infected plants after T-DNA integration (Veena and Taylor 2007). Which bacterial strain is suited best for the generation of composite plants has to be investigated individually for any plant species. Therefore, we compared four *A. rhizogenes* subtypes for their potential to induce transgenic poplar roots. To overcome problems with autofluorescence of poplar roots and in particular of ectomycorrhizas, we tested a peroxisome-targeted fluorescent protein approach to follow root transformation in vivo. Properties of ectomycorrhizas formed by transgenic roots of composite plants were analyzed in terms of anatomy, plant and fungal sugar content (as indicator for mycorrhizal physiology; Hampp et al. 1995, 2000), as well as gene expression. Here, we used an ectomycorrhiza-induced poplar SWEET gene as molecular marker for the formation of functional ectomycorrhizas (Nehls and Bodendiek 2012). SWEET proteins were characterized as sugar facilitators and are supposed to function as sugar efflux carriers in biotrophic interactions (Chen et al. 2010; Eom et al. 2015), including ectomycorrhizas (Nehls and Bodendiek 2012).

## Materials and methods

### Biological material

*Populus tremula* × *tremuloides* (clone T89), *P. tremula* × *alba* (INRA clone no. 717.1B4), and *P. trichocarpa* (Nisqually 1) plants were grown under sterile conditions on MS6 (Murashige and Skoog 1962) or McCown woody plant (Lloyd and McCown 1980; only *P. trichocarpa*) medium (both obtained from Duchefa Biochemie, Haarlen, The Netherlands); in a growth chamber (18 °C, 16 h, 80 µmol photons m^−2^ s^−1^ illumination). For propagation of the plants, shoot cuttings were used.

The following *A. rhizogenes* strains were analyzed for their capability to form poplar composite plants: 1724, a mikimopine strain (Shiomi et al. 1987); K599, a cucumopine strain (Daimon et al. 1990); 8196, a mannopine strain (Hansen et al. 1991); and 15834, an agropine strain (Veena and Taylor 2007). All *A. rhizogenes* strains were grown on solid CPY medium at 28 °C, supplemented with 50 mg l^−1^ kanamycin (Duchefa Biochemie) if necessary.

### Generation of composite poplars and ectomycorrhiza synthesis

*Agrobacterium rhizogenes* strains were transformed according to (Holsters et al. 1978) and grown on CPY agar plates supplemented with Kanamycin (50 mg/l) for up to 2 days at 28 °C until colonies appeared.

The cut surface of about 4-week-old sterile *P. tremula* × *alba* cuttings was dipped into a transformed agrobacterial colony grown on agar plates, poked about 4 mm deep into MS agar in a Petri dish, and incubated at 22 °C with 12 µmol photons m^−2^ s^−1^ illumination (16 h day–night rhythm) for 3 days. The cuttings were then transferred to fresh MS6 agar plates supplemented with carbenicilin (1.18 mM, Duchefa Biochemie) and cefotaxime (0.52 mM, Duchefa Biochemie) to restrain agrobacterial growth and cultivated as described before until transgenic roots reached a length of about 1–2 cm. These plants and non-inoculated control plants were further used for ectomycorrhizal formation under axenic conditions according to Hampp et al. (1996) with some modifications (see below) using *Amanita muscaria, Laccaria bicolor, Paxillus involutus*, or *Pisolithus tinctorius* as ECM fungal partner.

For mycorrhization, fungal mycelia were pregrown on modified MMN agar plates (Kottke et al. 1987) containing 50 mM glucose as sole carbon source at 18 °C for about 6 weeks. Six pieces of the growing mycelial front with about 5 mm edge length were transferred to fresh cellophane covered MMN agar plates containing 10 mM glucose and incubated at 18 °C in the dark for about 4 weeks. When the mycelium around the agar plugs reached a diameter of 2–3 cm, the membrane was transferred to fresh MMN medium having reduced nitrogen content (0.4 mM) and no carbon source. Poplar composite plants were transferred to these agar plates, such that the root system was placed inside the petri dish next to the mycelium, while the shoot remained outside (Hampp et al. 1996). Petri dishes were sealed with Parafilm and silicon grease and kept in a vertical position in a miniature greenhouse which provided high humidity at 18 °C. The root system was kept in dark by sheets of black paper covering the dishes, while the shoots were illuminated (16 h light–dark cycle and 80 µmol photons m^−2^ s^−1^). Ectomycorrhizas were formed after about 2–3 months.

### Microscopic inspection

Root formation of composite plants was followed by epifluorescence microscopy (Mz10F, Leica Microsystems, Wetzlar, Germany). Illumination was performed by UV light using a LQ-HXP 120 (Leistungselektronik JENA GmbH, Jena, Germany) as light source. The YFP filter set (excitation 510–520 nm, emission 540–560 nm) was used and images were taken using a video camera (DFC425 C, Leica).

Sections of non-infected fine roots and ectomycorrhizas were prepared using a vibratome (VT1000S, Leica). Fine roots and mycorrhizas were harvested using forceps (DuPont No. 5, Wilmington, USA) and embedded in 200 µl of preheated (60 °C) 4% water agarose. The root material was quickly transferred into a pressure vial (Dobner 2003) to remove air bubbles in the fungal mycelium surrounding the infected fine root (fungal mantle) and kept there until the agarose was completely solidified. Upper and the lower parts of agarose blocks were cut prior to fixation on the carrier using preheated agarose. About 100 µm thick cross and longitudinal sections were generated.

YFP expression was visualized in entire roots/mycorrhizas as well as in sections by confocal laser scanning microscopy (LSM 780, Carl Zeiss, Göttingen, Germany) using an argon laser (488 and 514 nm) with a primary beam splitter mirrors (488/514 nm) and a band-pass detector (sYFP detection window: 520–550 nm).

### Analysis of gene expression by quantitative RT-PCR (q-PCR)

Total RNA was isolated from *P. tremula* × *alba*/*A. muscaria* ectomycorrhizal or non-mycorrhizal fine roots using the NucleoSpin RNA Plant kit (Macherey-Nagel, Düren, Germany) according to the manufacturer’s instructions (including DNAse treatment). RNA integrity was proven by agarose gel electrophoresis and RNA content was quantified by photometry and image analysis. Aliquots containing 60 ng of total RNA were used for first-strand cDNA synthesis in a total volume of 20 µl, containing 50 pmol oligo-d(T)18-primer (Eurofins-MWG-Operon, Ebersberg, Germany), 0.5 μl RNAse inhibitor (RiboLock™ RNAse, 40 U/µl, Thermo Fisher Scientific), and 200 U Superscript II RNAse H-Reverse Transcriptase (Thermo Fisher Scientific) according to the manufacturer’s instructions. After synthesis, 30 µl of 5 mM Tris/HCl, pH 8 were added, and aliquots were stored at − 80 °C.

*PtaSWEET1*-specific primers (TTGGGCTACTTGGTGGTGACC, GATACCCATCTGCATTGACTTAAC) were designed, such that the reverse primer was located within the non-coding 3′ region. As references, gene-specific primers of *ubiquitin* (Brunner et al. 2004) or *actin* (Langer et al. 2004), which have been extensively used in previous publications (Selle et al. 2005; Ehlting et al. 2007; Escalante-Pérez et al. 2008; Willmann et al. 2014), were used as reference gene to calibrate the mRNA content.

Quantitative PCR was performed using 10 µl Q-PCR-Master mix (Thermo Fisher Scientific), 0.5 µl cDNA, and 10 pmol of each primer in a Light Cycler 480 System (Roche, Basel, Switzerland). PCR was always performed in triplicate.

Two independent cDNA synthesis reactions of two different root and mycorrhizal batches (obtained from at least four independent plants) were used for analysis of *PttSweet1c* expression in roots of control and composite plants. For determination of PCR efficiency, dilution series of each gene were prepared and used as template. The corresponding PCR efficiencies were calculated by the Light Cycler 480 software package (Version 1.5, Roche).

### Analysis of soluble sugars

Soluble sugars were quantified according to Schaeffer et al. (1995) with some modifications (see below). Freshly isolated mycorrhizas of transgenic and non-transgenic plants (two batches of six plants each) were isolated from agar plates, pooled and snap frozen in liquid nitrogen. Grinding to a fine powder was performed under liquid nitrogen using a pestle and a 1.5 ml Eppendorf tube as morter. The powder was freeze dried under vacuum and aliquots of about 0.5 mg were transferred into fresh 1.5 ml Eppendorf tubes in a conditioned room (40% humidity, 18 °C). Extraction of soluble sugars was performed with a total volume of 400 µl methanol using a pestle and a 1.5 ml Eppendorf tube as morter. After incubation of the suspensions for 1 h at 60 °C, the samples were centrifuged (50 min at 20 °C and 20,000*g*) and 320 µl of the supernatant were transferred into fresh 1.5 ml tubes. Methanol was evaporated under vacuum, soluble sugars were dissolved in 90 µl higly pure water, and aliquots were applied to a Dionex DX 500-liquid chromatography system (Thermo Fisher Scientific GmbH, Dreieich, Germany) containing a CarboPack PA10 (Thermo Fisher Scientific). Sugar quantification was performed using a Pulsed Amperometric Detector (ED40, Thermo Fisher Scientific) and calibration standards.

## Results

### Transgenic root induction by *A. rhizogenes*

**Fig. 1:**
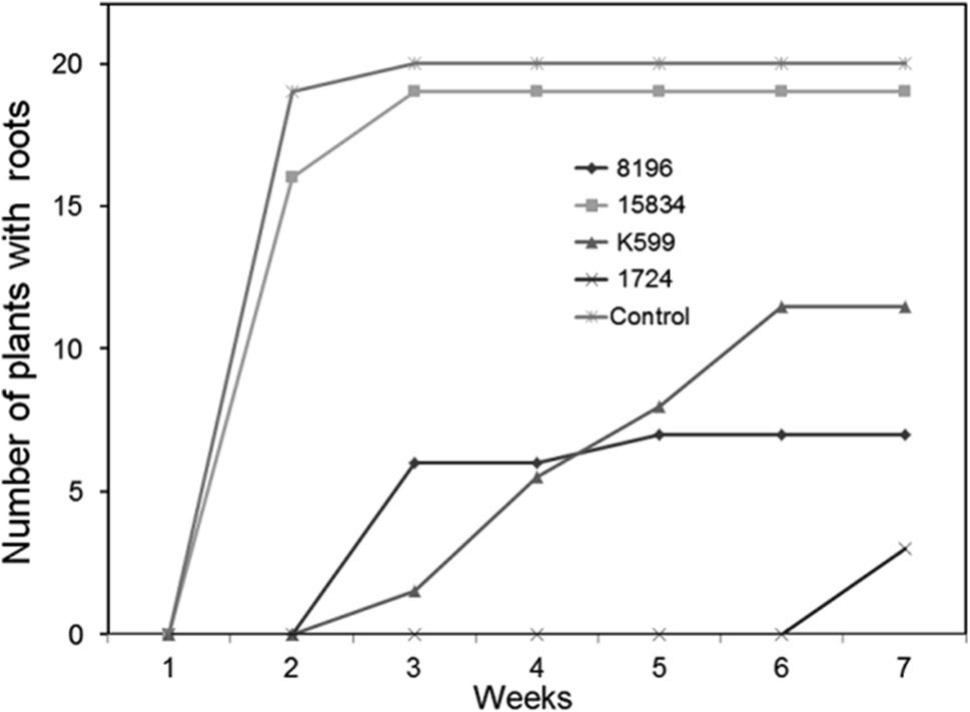
Kinetics of root formation of poplar cuttings inoculated with different *A. rhizogenes* strains. Root formation by poplar cuttings inoculated with one of the following *A. rhizogenes* strains 15834, K599, 8196, 1724, or water (control) was followed over a time period of 7 weeks. Shown is the percentage of plants that had formed at least a single root at the given timepoint. A total of 20 cuttings each were used for the analysis

**Fig. 2:**
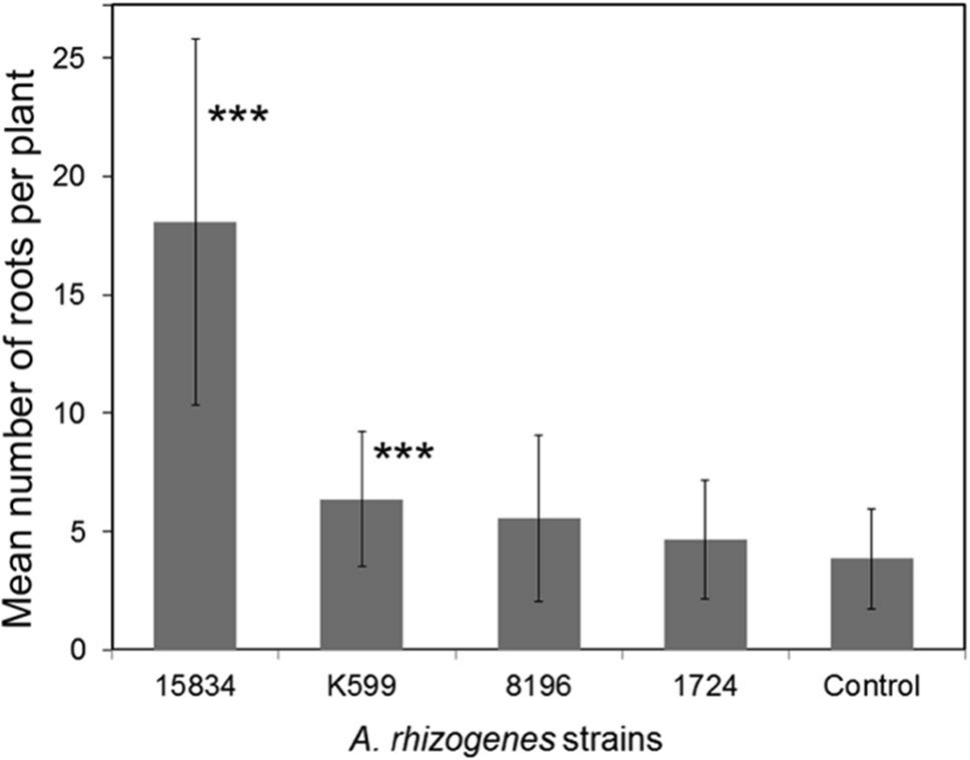
Mean number of roots formed per plant. Plants from Fig. 1 were assessed for the number of roots that were formed per poplar cutting after 7 weeks of inoculation with the respective agrobacterial strain or distilled water (control). Plants that did not form roots at all were omitted from this analysis. Shown are mean values and standard errors. The number of roots per plant differed significantly (student *t* test) from that of control plants (20 plants) for 15834 (*p* value: 2.8 × 10^−9^, 19 plants) and K599 (8.13 × 10^−4^, 12 plants) but not for 8196 (*p* value: 0.132, 7 plants) and 1724 (*p* value: 0.546, 3 plants)

**Fig. 3:**
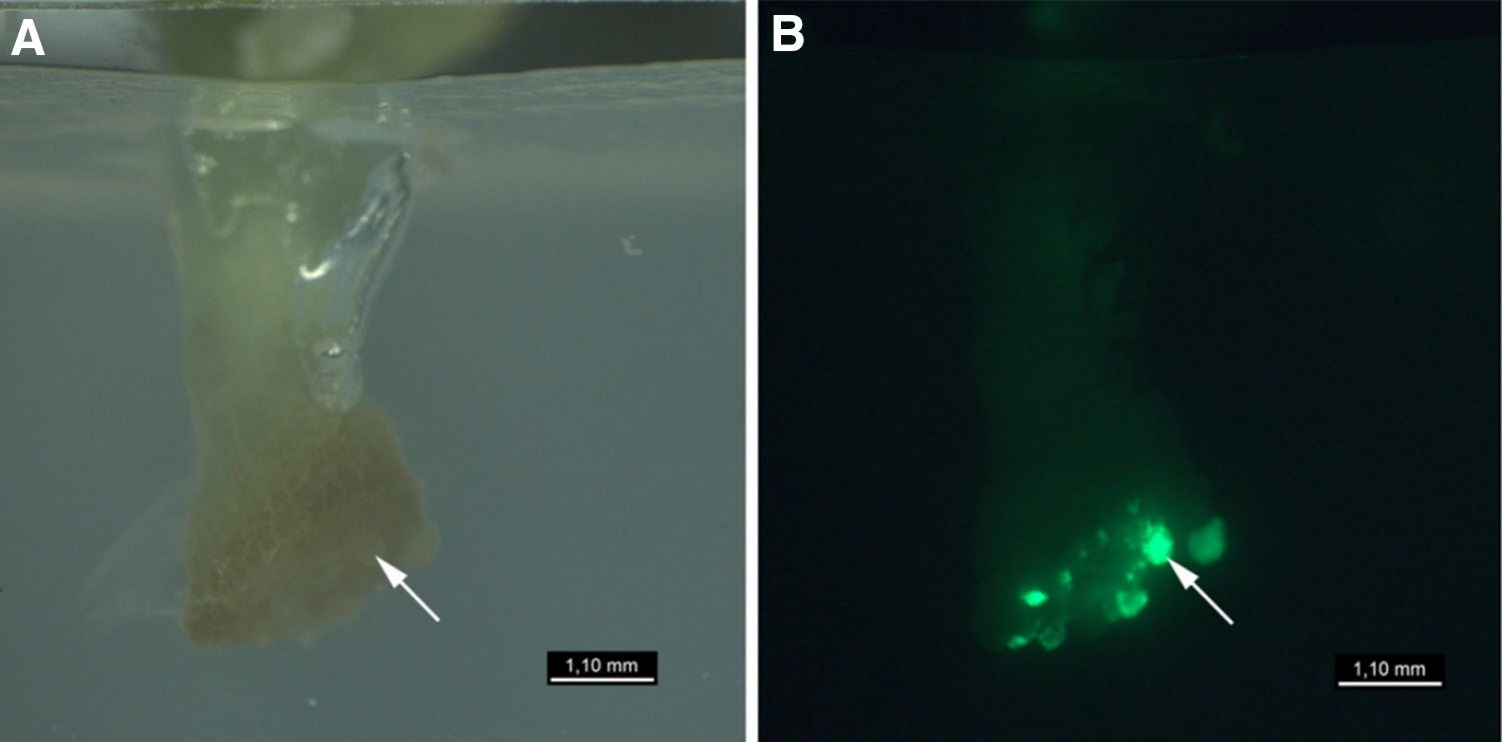
Microcallus formation at the shoot surface of cuttings inoculated with *A. rhizogenes* strain K599. The formation of microcalli at the surface of *P. tremula* × *alba* cuttings was visualized 3 weeks after inoculation with pBIN19-YFPPTS1 transformed *A. rhizogenes* strain K599 by microscopy: **A** transmission light, **B** UV light (YFP filter set). Labelled by an arrow is a transgenic microcallus from which root formation is initiated

We have tested four different *A. rhizogenes* strains for their capacity to induce and transform shoot based roots using sterile, about 1-month-old *P. tremula* × *tremuloides* (data not shown) and *P. tremula* × *alba* cuttings. Both hybrid poplars revealed similar results. Except for the agropine *A. rhizogenes* strain 15834 that revealed a kinetic similar to that of the water control, root formation was retarded when plants were inoculated with agrobacteria (Fig. 1). Especially, the mikimopine strain 1724 revealed a large proportion of plants (85%), where no roots were formed even after 7 weeks. For plants, where roots were formed after seven weeks of inoculation, the root number per plant did not differ significantly between water control (3.85 ± 2.1) and plants inoculated with the *A. rhizogenes* strains 1724 (4.67 ± 2.52) or 8196 (5.56 ± 3.51) (Fig. 2). In contrast, plants inoculated with the cucumopine strain K599 revealed a slight (6.37 ± 2.85) but highly significant increase in root number, while those inoculated with 15834 revealed many more (18.06 ± 7.74) roots per plant. Except for control and 15834 (agropine strain) mediated composite plants (data not shown), poplar cuttings inoculated with K599 (cucumopine strain), 8196 (mannopine strain), and 1724 (mikimopine strain) developed microcalli at the infected shoot surface prior to root formation (an example for K599 is shown in Fig. 3).

To visualize *Agrobacterium*-mediated stable T-DNA transmission from a binary vector into the plant genome, all agrobacterial strains were transformed with the binary vector pBIN19-YFPPTS1 (Nowak et al. 2004) prior to plant infection. This vector contains a marker cassette that allows a strong, constitutive in planta expression of a peroxisome-targeted yellow fluorescent protein (YFP) as a consequence of successful plant transformation. Out of the four tested agrobacterial strains, only K599 reproducibly allowed easy in planta YFP signal detection by epifluorescence microscopy for both hybrid poplars (in Fig. 3B an example for *P. tremula* × *alba* is shown), while for *P. trichocarpa*, no fluorescent roots were obtained at all. Because inoculation of poplar cuttings with the agrobacterial strain K599 showed furthermore only minor retardation in root formation and a weak tendency for the hairy root phenotype (e.g., formation of a moderate increased root number), this strain was selected for further analysis.

### Induction and growth properties of roots on sucrose containing medium

The standard rooting medium for poplar cuttings contains 1% sucrose, which has been shown to be a potent root morphogen in *Arabidopsis*. We have, therefore, compared formation and growth properties of roots on sucrose containing or free agar plates using non-inoculated (control) and K599 inoculated cuttings.

**Fig. 4:**
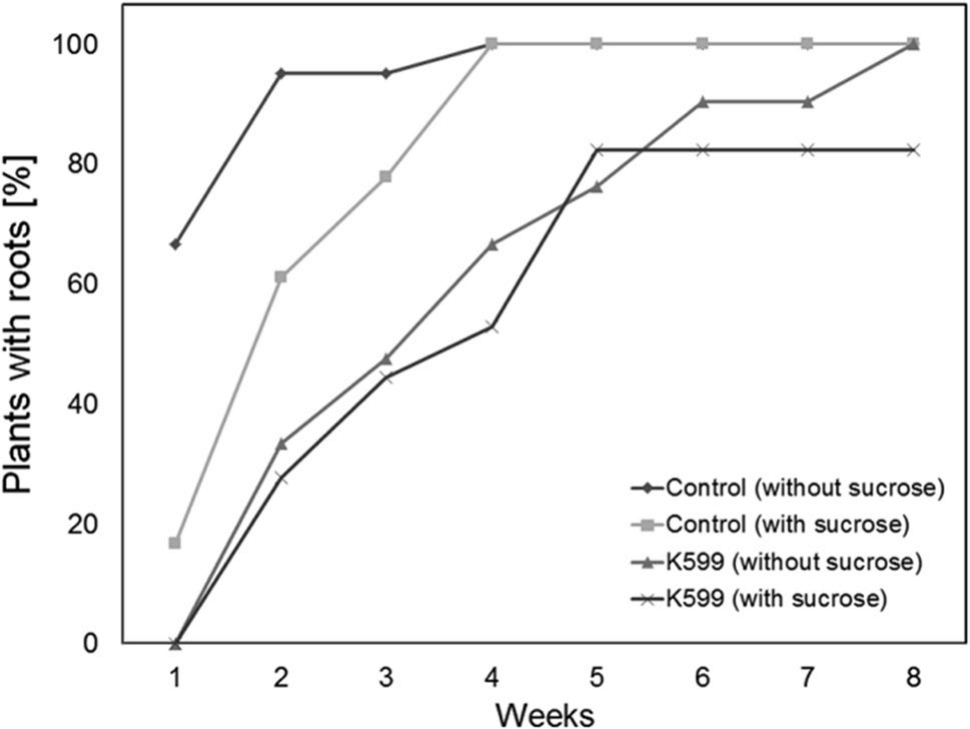
Effect of sucrose on the root formation of poplar cuttings inoculated with *A. rhizogenes* strain K599 or water. Root formation was assessed once a week for a period of 8 weeks after inoculation of poplar cuttings (*n* = 20, each) with the *A. rhizogenes* strain K599 or water (as control). Shown is the percentage of cutting that revealed at least one root per plant at a given timepoint

When control cuttings were incubated in sucrose-free medium, 67% revealed initial root formation after one week, while only 17% of the cuttings started rooting on sugar containing medium (Fig. 4). However, all control plants were rooted after 4 weeks, independently on whether they were grown on sucrose containing or free medium. In contrast, cuttings inoculated with K599 showed no obvious difference in root formation kinetics on sucrose containing or sucrose-free agar (Fig. 4). Furthermore, K599-inoculated poplars revealed a slightly larger number of rooted cuttings after 8 weeks of growth in the absence of sucrose (Fig. 4).

When grown on sucrose containing medium, control plants showed a highly significant (*p* = 0.0004) larger root number after 8 weeks (9.6 ± 3.3 versus 6.2 ± 1.9), while K599 treated cuttings revealed only a not significant (*p* = 0.38) tendency for a larger root number (7.4 ± 4.2 versus 5.7 ± 2.3).

Sucrose also had a severe impact on root branching for both control- and K599-treated cuttings. The presence of sucrose in the growth medium significantly (*p* = 1.7 × 10^−9^ and 7.32 × 10^−6^, respectively) elevated the number of branches per root (control cuttings: 11 ± 7.1 versus 0.5 ± 0.8; K599-treated cuttings: 8.3 ± 7 versus 2.0 ± 2.4).

### Root cotransformation efficiency

To determine the efficiency by which the binary vector located T-DNA was transmitted into shoot-derived poplar roots, cuttings were inoculated with transgenic K599 harboring a peroxisome-targeted YFP under the control of a constitutive promoter. Roots were investigated after 8 weeks of inoculation for YFP expression by epifluorescence microscopy. 50 out of a total of 55 composite plants showed at least one root that expressed the YFP marker and 88.1% of all roots that were formed showed YFP expression, demonstrating the success of the composite plant strategy to obtain transgenic poplar roots. In addition to pBin19 (Bevan 1984), we have also tested binary vectors with pGreenII (Hellens et al. 2000) background for K599-based poplar transformation, revealing similar transformation efficiencies (data not shown).

### Cell wall autofluorescence of poplar roots may interfere with fluorescent protein signal detection

**Fig. 5:**
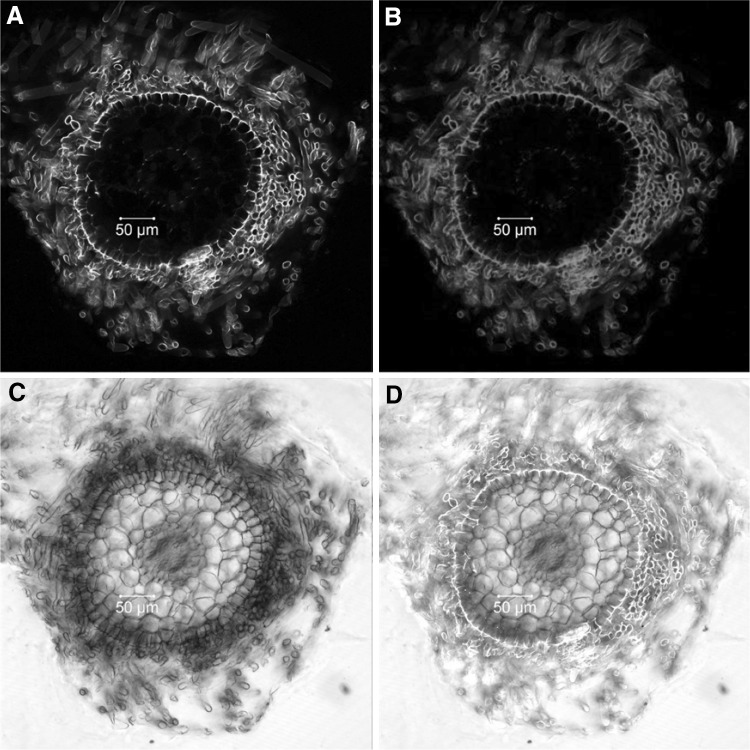
Autofluorescence of poplar fine roots. Radial sections of non-transgenic poplar fine roots were obtained from fresh root material and analyzed by laser scanning microscopy (excitation: argon laser, 488 nm, 10% power). Shown are (in false color) the YFP channel (**A**, detection range 510–560 nm), the ds red channel (**B**, detection range 560–650 nm), the transmission light channel (**C**, TMP), and an overlay of the three images (**D**). (Color figure online)

**Fig. 6:**
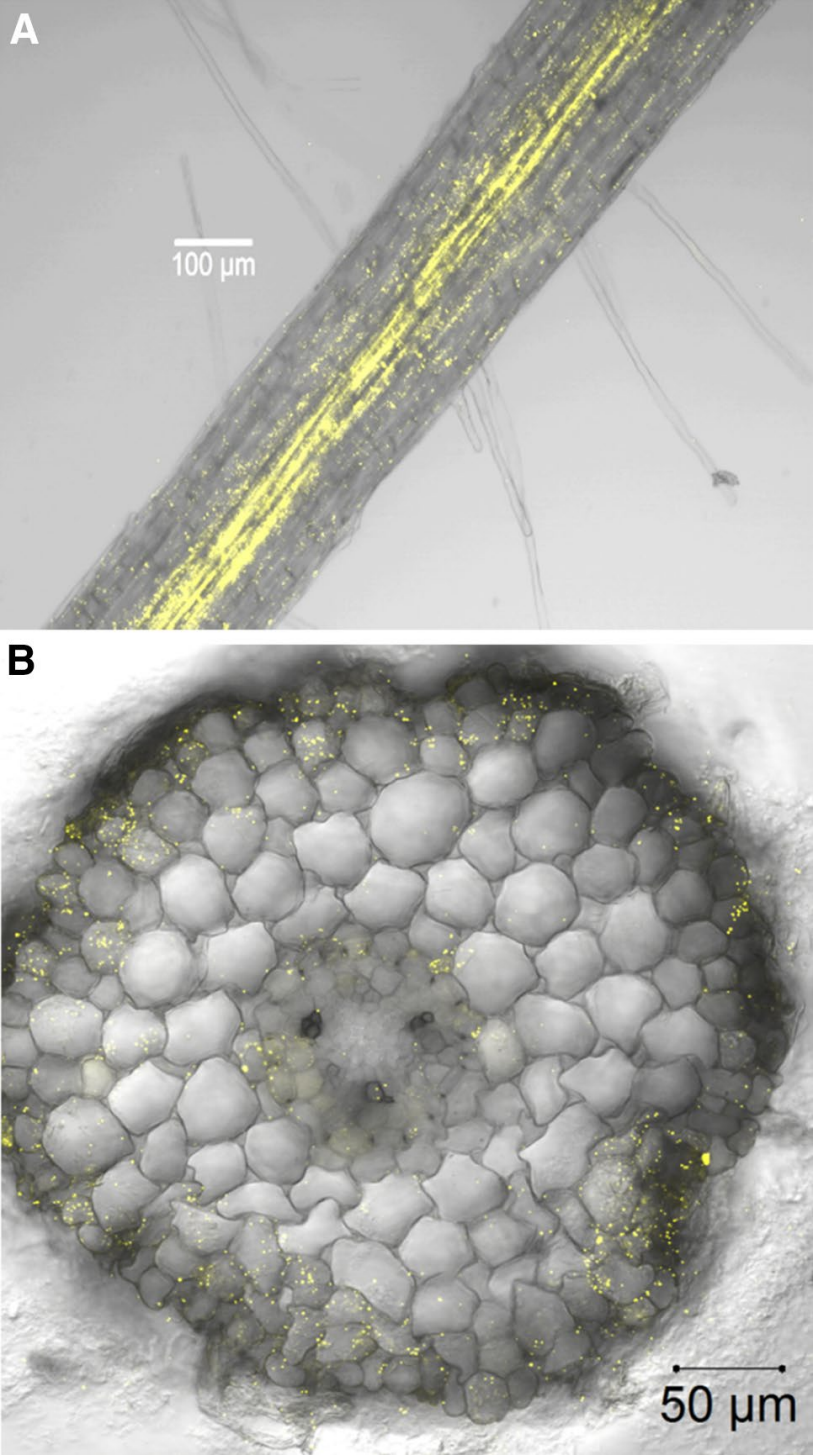
Peroxisome located YFP fluorescence of a transgenic root mediated by *A. rhizogenes* transformation. 6-week-old *A. rhizogenes* (K599) mediated transgenic roots of composite *P. tremula* × *alba* plants were investigated for peroxisome-targeted yellow fluorescent protein distribution. Whole mounts as well as cross sections were visualized by laser scanning microscopy (excitation: argon laser, 488 nm, 2% power). Peroxisomes are shown in yellow (false colors). Images are overlays of the YFP channel (detection range 510–560 nm) and the transmission light channel. Shown are in **A** a whole mount view and in **B** a cross section of an YFP expressing transgenic poplar root. (Color figure online)

Strong cell wall autofluorescence was observed in non-mycorrhized (Fig. 5) and mycorrhized (Fig. 8) poplar fine roots exposed to laser intensities of 5% power and more. This autofluorescence covered a wide emission spectrum (500–650 nm), independent on whether 458, 488, or 561 nm laser lines are used for excitation. For non-infected fine roots, autofluorescence became much weaker at laser intensities below 2%, allowing a better discrimination of the specific YFP signal (Fig. 6A, B). However, in ectomycorrhizas, cell wall autofluorescence was much stronger (especially in the Hartig net area, Fig. 8). Because some peroxisomes are always located in a given distance to the cell wall, a clear YFP signal recognition was still feasible (yellow dots in Fig. 8).

No obvious chimera roots, revealing fluorescent and non-fluorescent branches, were ever observed in poplar composite plants (data not shown). Furthermore, cross sections along transgenic roots (from tip to base) indicated YFP fluorescent peroxisomes in cells of all root tissues (rhizodermis, cortex, endodermis, stele; an example of a cross section is shown in Fig. 6B).

### Ectomycorrhiza formation and function

**Fig. 7:**
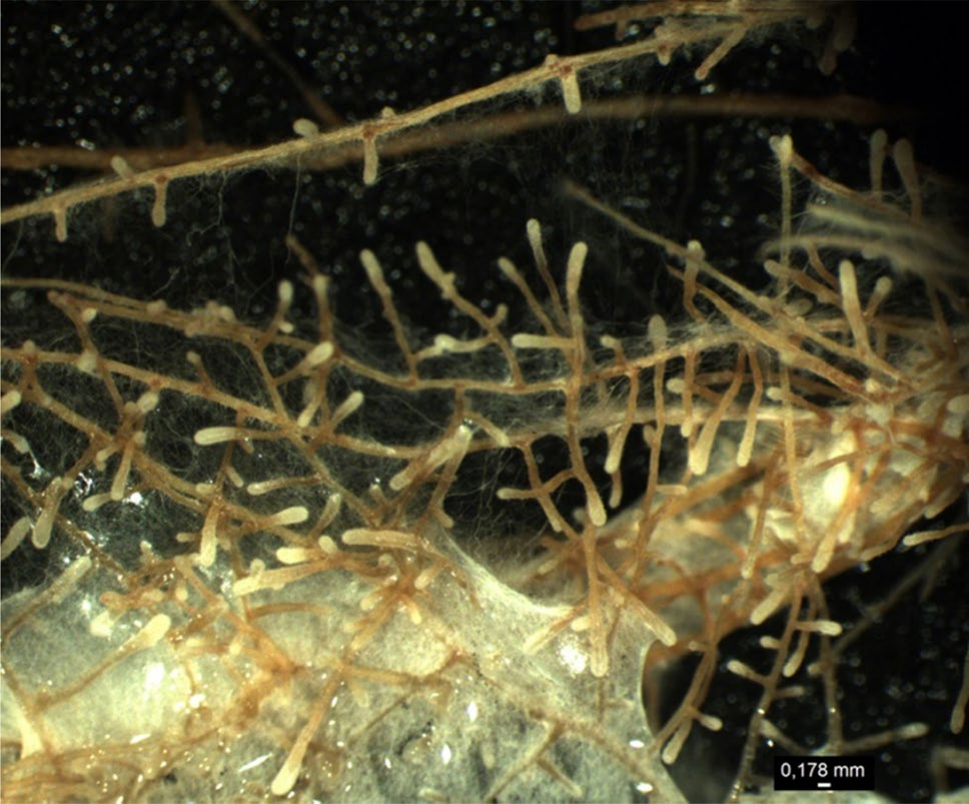
Typical ectomycorrhizas formed by roots of a transgenic composite poplar. *P. tremula* × *alba* cuttings were inoculated with transgenic *A. rhizogenes* (K599) expressing a peroxisome-targeted YFP under the control of a constitutive promoter. Composite poplar plants were used for inoculation with *A. muscaria* as ectomycorrhizal fungal partner when newly formed roots reached a length of about 2 cm. Shown is a section of the root system of a composite plant after 3-month of inoculation

**Fig. 8:**
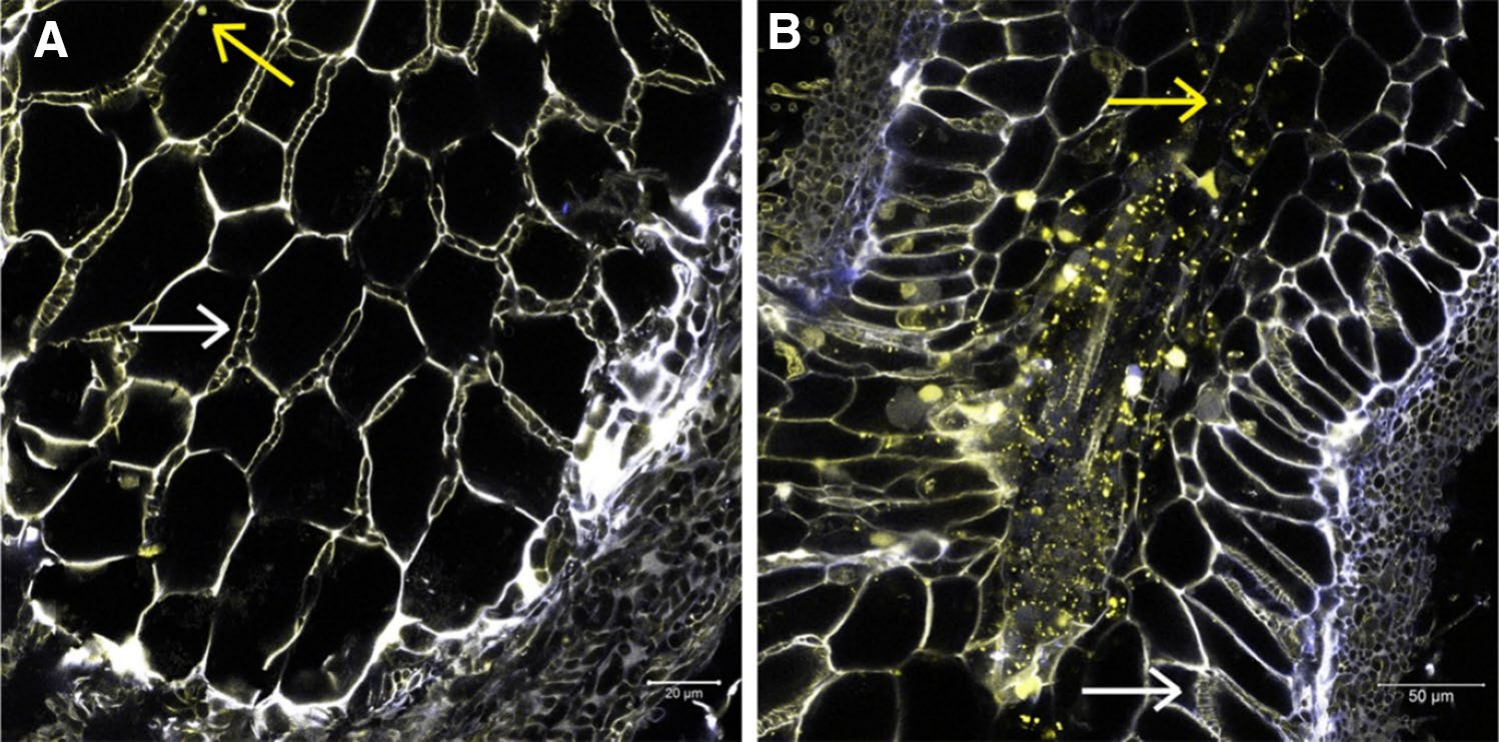
Longitudinal sections through a typical ectomycorrhiza. Cell walls of fungal hyphae and root cells were visualized by their autofluorescence after illumination with an argon laser beam (488 nm, 10% power). Shown are overlays of the YFP channel (in yellow, detection range 510–560 nm) and the ds Red channel (in blue, detection range 560–650 nm). Shown are longitudinal sections through **A** cortex layer of an infected fine root and **B** deeper layer of another root at a lower magnification, where the central cylinder (yellow arrow) can be seen in addition to the Hartig net (white arrow) and the root cortex. Examples, where fungal hyphae can be easily visualized within the Hartig net are marked by white arrows. Yellow dots: peroxisome-targeted YFP (examples are indicated by yellow arrows). (Color figure online)

*Agrobacterium rhizogenes*-mediated transformed poplar roots were able to form ectomycorrhizas with *A. muscaria* (Fig. 7), *L. bicolor, P. involutus*, and *P. tinctorius* (data not shown) under sterile conditions in a time frame of about 2–3 months after inoculation. Longitudinal sections through ectomycorrhizal roots (Fig. 8A, B) revealed typical mycorrhizal structures with elongated rhizodermal cells in the Hartig net area and a characteristic fungal sheath, composed of several layers of fungal mycelium covering the infected fine root (Fig. 8B).

To test the functionality of ectomycorrhizas formed by the transgenic roots, two approaches were followed: (a) determination of plant- and fungus-specific sugars and (b) transcript levels of the ectomycorrhiza-specific induced poplar gene *PtaSWEET1*.

**Fig. 9:**
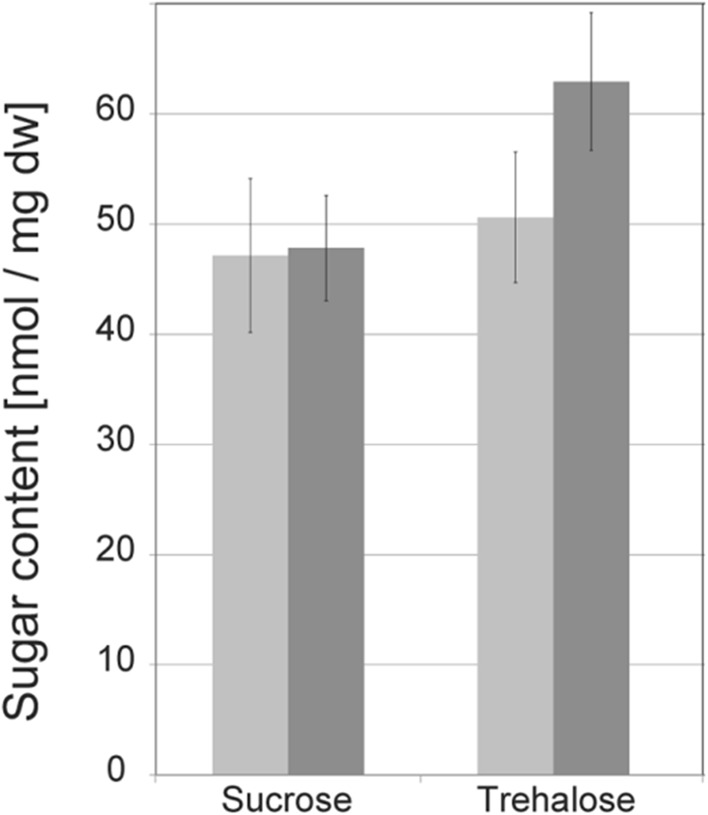
Sugar content in mycorrhizas of transgenic and non-transgenic plants. Mycorrhizas were harvested from transgenic and non-transgenic roots of *P. tremula* × *alba* plants grown for about 3 months in Petri dishes with *A. muscaria* as ECM fungal partner. Shown is the sugar (trehalose, sucrose) content of mycorrhizas of non-transgenic (light gray) and transgenic (dark gray) roots in nmol/mg dry weight. *P* values comparing sugar content of mycorrhizas of transgenic and non-transgenic roots are as follows (Student’s *t* test): sucrose: *p* = 0.85, trehalose: *p* = 0.006

**Fig. 10:**
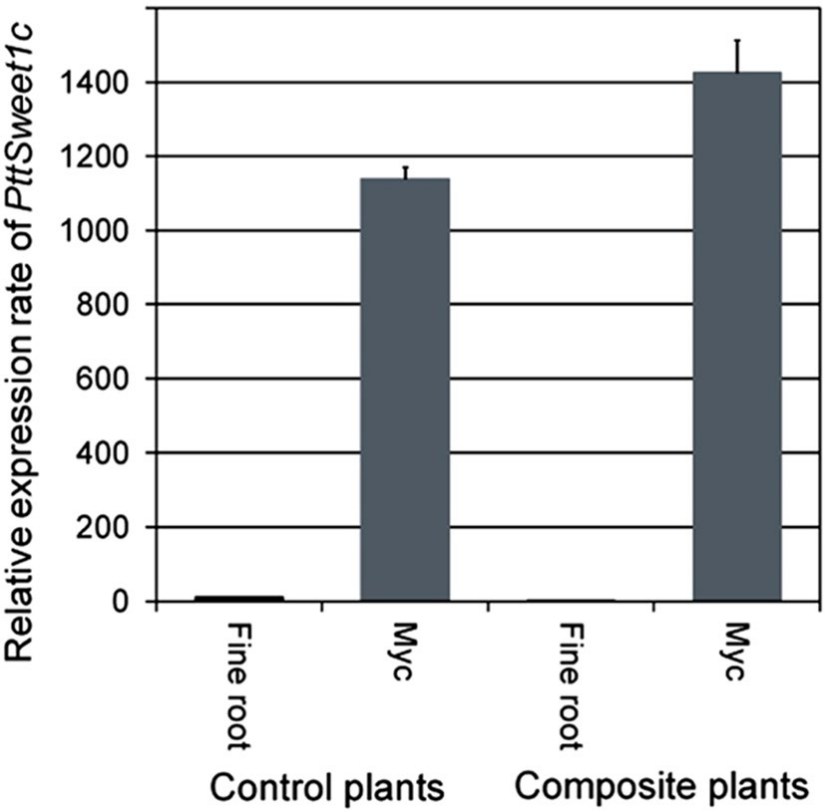
*PtaSWEET1* expression in ectomycorrhizas and non-mycorrhized fine roots of transgenic composite and non-transgenic control plants. Fine roots and mycorrhizas were harvested from transgenic composite and non-transgenic *P. tremula* × *alba* plants grown for about 3 months in Petri dishes in the presence or absence of *A. muscaria* as ECM fungal partner. First-strand cDNA (as template) together with gene-specific primers was used for expression analysis (quantitative RT-PCR). Shown are mean values and standard errors of CT ratios of *PtaSWEET1* and *ubiquitin* (comparable results were obtained by using actin as reference)

- Sucrose (plant origin) and trehalose (fungal origin) contents of ectomycorrhizas of transgenic and non-transgenic poplar roots were analyzed by liquid chromatography. While no significant difference in sucrose content was observed, trehalose content was significant higher (about 24%) in ectomycorrhizas of transgenic roots (Fig. 9).
- Ectomycorrhizal marker gene (*PtaSWEET1*) expression was analyzed by q-PCR. Similar to wild-type roots, an ectomycorrhiza-specific induction of *PtaSWEET1* expression was also observed in mycorrhizas of transgenic composite plants (Fig. 10). Interestingly, like the fungal sugar content, *PtaSWEET1* transcript levels were also about 25% higher in mycorrhizas of transgenic roots.«

## Discussion

Four different *A. rhizogenes* strains, belonging to different opine types, were analyzed for their suitability for poplar root transformation. Apart from the agropine type (strain 15834), all other strains induced extended microcallus formation at the cut surface of poplar cuttings prior to rooting. As a consequence, retarded root formation was observed. Furthermore, microcallus formation complicated plant handling upon ectomycorrhiza synthesis as root harboring microcalli were fragile. Nevertheless, even when forming microcalli, strain K599 turned out to be the most suitable for a reproducible induction of transgenic roots by poplar cuttings. This is in agreement with a systematic investigation of the usability of *A. rhizogenes* strains for plant root transformation by Porter and Flores (1991), who also stated strain K599 as being the most efficient. In contrast to hybrid poplars, we were not able to observe transgenic root formation on *Populus trichocarpa* cuttings, which is in agreement with many unsuccessful attempts of *A. tumefaciens*-based *P. trichocarpa* transformation (e.g., Böhlenius et al. 2006).

Sucrose containing agar medium is routinely used for rooting of poplar cuttings. Sucrose is, however, known a potent plant root morphogen (Freixes et al. 2002; Lee-Ho et al. 2007). As *A. rhizogene*s-based transformation frequently results in modulation of root phytohormone response, such a sugar effect might enhance the hairy root phenotype of composite plants. Indeed, sucrose containing agar medium increased root branching. However, this phenomenon was observed for both non-transgenic and transgenic roots and was not more pronounced in transgenic roots. As rooting of K599 inoculated plants was even a bit better without sucrose, we routinely omitted sucrose from the rooting medium.

Even when poplar transformation efficiency was in the range of other plants (Porter and Flores 1991), a distinct number of newly formed roots showed a slight hairy root phenotype but no fluorescent marker expression, indicating that the T-DNA of the binary vector was not transmitted together with the T-DNA of the Ri plasmid. If binary vector based T-DNA does not allow direct visualization of successful root transformation (e.g., in case of RNAi or overexpression approaches), examination of a much larger root number would be the consequence. A strategy to solve this problem is the additional integration of a visual marker cassette into the T-DNA region of the binary vector. Peroxisomal targeted fluorescent proteins (as used in this work) would be ideally suited as they allow an easy in planta detection of transgenic roots without severe disturbance of root physiology.

Peroxisomal targeted fluorescent proteins also helped to cure a general problem of poplar fine roots and in particular ectomycorrhizas: cell wall autofluorescence, which covers a wide emission range and hence cannot be simply eliminated technically. Rhizodermal and root cortical cells comprise large central vacuoles. As a consequence, the cytoplasmic layer has a thickness of about that of the cell wall, making detection of cytoplasm located fluorescent proteins rather difficult. In non-mycorrhized poplar roots (but not ectomycorrhizas), argon laser intensities below 2% eliminate the autofluorescence problem, but do request a strong specific fluorescence signal. Targeting of fluorescent proteins into peroxisomes leads to protein enrichment in small cellular compartments and thus strong fluorescence signal intensities. Peroxisomes are, furthermore, frequently found far away from cell walls, e.g., in cytoplasmic tubes crossing the central vacuole. In cases, where cell wall autofluorescence is still visible even at low laser intensities, e.g., in ectomycorrhizas, peroxisomes that are located in a given distance from the cell wall allow specific and highly sensitive fluorescence signal detection.

Every root formed by a composite plant could be the result of an independent transformation event (Costantino et al. 1983). Different copy numbers or positional effects of genome integrated T-DNAs (e.g., Collier et al. 2005) would result in varying gene expression levels in individual roots. Indeed, independent roots of single composite poplars showing differences in YFP fluorescence intensity were observed in this work (data not shown). However, the conventional transgenic plants also show such variation, resulting in the necessity to analyze a given number of independent transformants. Whether or not individual root transformation events limit composite plant application is depending on the question to be addressed. Based on fluorescent protein expression and microscopy, we successfully performed promoter analysis of ectomycorrhiza-induced genes with composite poplars (Nehls et al. unpublished). For such analysis, only minor amounts of roots/ectomycorrhizas are requested and analysis of single roots is feasible even in axenic culture (Petri dish system) with a limited amount of root material. For biochemical analysis, however, small root systems can be problematic. In such cases, transfer of plants into pot culture might be a solution to obtain sufficient root material. Pot culture is, however, hardly feasible under sterile conditions. An alternative strategy is thus pooling of root material of different plants prior to analysis. Of course, individual differences will be no longer recognized by this approach, but for many questions, e.g., assessment of the impact of a manipulation, an average picture is sufficient. We successfully followed this strategy for many years when pooling ectomycorrhizas from root systems of different plants prior to analysis. Individual ectomycorrhizas can be rather variable, e.g., in terms of sugar content (Nehls, unpublished). Batchwise analysis gives, however, similar results as analysis of individuals and calculation of means, if sufficient large numbers of mycorrhizas were used (Fajardo López et al. 2007; Nehls, unpublished).

Using the later strategy, we were able to compare ectomycorrhizal sugar content and marker gene expression in infected transgenic and non-transgenic fine roots. As fungal mycelia and plant root cells are inseparably linked in ectomycorrhizas, only organism-specific metabolites can be allocated to a given partner in symbiosis. With regard to sugars, sucrose (plant origin) and trehalose (fungus origin) fulfil this criterion. Whereas plants are capable in trehalose biosynthesis, this sugar is hardly detectable in plant tissues (Müller et al. 1999) and trehalose content of ectomycorrhizas is thus assumed to be solely of fungal origin (e.g., Fajardo López et al. 2007). While sucrose content was indistinguishably between ectomycorrhizas of non-transgenic and transgenic fine roots, trehalose content was slightly but significantly higher (24%) in mycorrhizas of transgenic roots. This indicates that fungal sugar support is somewhat better in transgenic roots of composite poplars. Furthermore, transcript levels of polar *PtaSWEET1* were also about 25% higher in ectomycorrhizas of transgenic roots, which is in line with the elevated fungal trehalose content. SWEET proteins are characterized as sugar facilitators and are supposed to function as sugar efflux carriers in certain biotrophic interactions (Chen et al. 2010). In poplar ectomycorrhizas, distinct members of the SWEET gene family (e.g., *PtaSWEET1*) are induced in a symbiosis-specific manner and are thus supposed to be responsible for fungal sugar support (Nehls and Bodendiek 2012; Nehls, unpublished). Increased *PtaSWEET1* transcript levels of composite poplar roots thus indicate elevated sugar efflux from root cells, resulting in better fungal carbon nutrition and, in a consequence, in increased trehalose content of ectomycorrhizal fungal mycelia.

The known consequence of Ri-plasmid-based T-DNA integration into the plant root genome is modulation in phytohormone (especially auxin) response (Christey 2001; Kiselev et al. 2007). As root transformation did not result in elevated *PtaSWEET1* transcript levels, such a variation in phytohormone response is not sufficient to control *PtaSWEET1* expression. However, similar to *A. rhizogenes*-based root transformation, fungal hyphae manipulate root phytohormone content upon ectomycorrhizal symbiosis, which has been frequently discussed as a central mechanism for influencing plant root morphology and function (Nehls et al. 1998; Vayssières et al. 2015). *A. rhizogenes* T-DNA triggered improvement of fungal sugar support in symbiosis thus indicates a direct link between root auxin content/susceptibility and fine tuning of fungal carbon support and will be further addressed in upcoming research.

## Summary

We have developed a protocol for generation of composite poplars that reduce the time demand for formation of transgenic plants from several months to a few weeks. While all investigated agrobacterial isolates were able to transform poplar roots, striking drawbacks were observed for three of them, making the strain K599 in our hands most suitable for poplar transformation. Transgenic root formation on non-transgenic, sterile cuttings was regularly induced. Such roots showed an only weak tendency for a hairy root phenotype but strong marker expression (fluorescence). In poplar fine roots but particularly in ectomycorrhizas, peroxisomal localization of the fluorescent signal leads to a sensitive in planta detection of gene expression and solves the problem of a broad cell wall autofluorescence. Finally, both fungal trehalose content and plant *PtaSWEET1* expression point towards elevated fungal carbon support in ectomycorrhizas of transgenic poplar fine roots.

### Author contribution statement

UN conceived and designed research and supervised DN, AD, and AH. DN, AD, AH, and UN conducted experiments; DN, AD, and UN analyzed data. UN, DN, and AD wrote the manuscript. All authors read and approved the manuscript.
